# New Therapeutic Insight into the Effect of Ma Huang Tang on Blood Pressure and Renal Dysfunction in the L-NAME-Induced Hypertension

**DOI:** 10.1155/2021/9980429

**Published:** 2021-07-13

**Authors:** Mi Hyeon Hong, Hye Yoom Kim, Youn Jae Jang, Se Won Na, Byung Hyuk Han, Jung Joo Yoon, Chang Seob Seo, Ho Sub Lee, Yun Jung Lee, Dae Gill Kang

**Affiliations:** ^1^Hanbang Cardio-Renal Syndrome Research Center, Wonkwang University, 460 Iksan-daero, Iksan, Jeonbuk 54538, Republic of Korea; ^2^College of Korean Medicine and Professional Graduate School of Korean Medicine, Wonkwang University, 460 Iksan-daero, Iksan, Jeonbuk 54538, Republic of Korea; ^3^Herbal Medicine Research Division, Korea Institute of Oriental Medicine, 1672 Yuseong-daero, Yuseong-gu, Daejeon 34054, Republic of Korea

## Abstract

In this study, we evaluated the effect of a traditional herbal formula, Ma Huang Tang (MHT), on blood pressure and vasodilation in a rat model of N^G^‐nitro‐L‐arginine methylester- (L-NAME-) induced hypertension. We found that MHT-induced vascular relaxation in a dose-dependent manner in rat aortas pretreated with phenylephrine. However, pretreatment of endothelium-intact aortic rings with L‐NAME, an inhibitor of nitric oxide synthesis (NOS), or 1H‐[1, 2, 4]‐oxadiazole‐[4, 3‐*α*]‐quinoxalin‐1‐one (ODQ), an inhibitor of soluble guanylyl cyclase, significantly abolished vascular relaxation induced by MHT. MHT also increased the production of guanosine 3′,5′-cyclic monophosphate (cGMP) in the aortic rings pretreated with L-NAME or ODQ. To examine the *in vivo* effects of MHT, Sprague Dawley rats were treated with 40 mg/kg/day L-NAME for 3 weeks, followed by administration of 50 or 100 mg/kg/day MHT for 2 weeks. MHT was found to significantly normalize systolic blood pressure and decreased intima-media thickness in aortic sections of rats treated with L-NAME compared to that of rats treated with L-NAME alone. MHT also restored the L-NAME-induced decrease in vasorelaxation response to acetylcholine and endothelial nitric oxide synthase (eNOS) and endothelin-1 (ET-1) expression. Furthermore, MHT promoted the recovery of renal function, as indicated by osmolality, blood urea nitrogen (BUN) levels, and creatinine clearance. These results suggest that MHT-induced relaxation in the thoracic aorta is associated with activation of the nitric oxide/cGMP pathway. Furthermore, it provides new therapeutic insights into the regulation of blood pressure and renal function in hypertensive patients.

## 1. Introduction

Hypertension is a major cause of cardiovascular diseases, such as heart failure, stroke, and myocardial infarction [1]. Adding to this, 10% mortality due to hypertension is caused as a result of renal dysfunction and failure. Systemic arterial hypertension is closely related to vascular endothelial cell dysfunction [2]. Endothelial cells are stimulated by releasing endothelium-dependent vasodilators or endothelium-derived relaxing factors. Nitric oxide (NO), which is generated in endothelial cells, activates soluble guanylyl cyclase (sGC) and increases the production of intracellular guanosine 3′,5′-cyclic monophosphate (cGMP) [3], which induces vascular relaxation. The impairment of the NO-cGMP pathway induces hypercholesterolemia, diabetes, and hypertension because of a decline in vascular relaxation [4, 5]. Nonmediated vascular relaxation is essential for the maintenance of vascular function. NO is also an important regulator of blood pressure, regional blood flow, vascular smooth muscle proliferation, platelet aggregation, and leukocyte adhesion [6]. Vascular activity regulation by the NO pathway is altered in various models of hypertension [7]. Of them, in this study, we used the N^G^‐nitro‐L‐arginine methylester (L-NAME) model to study the *in vivo* vascular response after chemical inhibition of endothelial NOS (eNOS) production.

Ma Huang Tang (MHT), mentioned in the Treatise on Febrile Disease of Zhang Zhongjing, has been used for thousands of years to treat many diseases, such as exogenous wind-cold, cough, and asthma [8]. MHT is a formulation derived from Ephedra (*Ephedra sinica*), Cinnamomi Ramulus (*Cinnamomum cassia*), Armeniacae Semen (*Prunus armeniaca*), and Glycyrrhizae Radix et Rhizoma Praeparata Cum Melle (*Glycyrrhiza uralensis*). Modern pharmacological research has shown that MHT exhibits therapeutic effects against inflammation, allergy, asthma, and hyperglycemia [8, 9] and is used to treat bronchitis, asthma, respiratory infection, hypertension, acute glomerulonephritis, and chronic renal failure [10]. However, the therapeutic effect of MHT on hypertension has not been investigated. Therefore, in this present study, we investigated the mechanism of MHT-induced vascular relaxation in rat aorta and its effect on blood pressure and renal function in rats with L-NAME-induced hypertension.

## 2. Experimental Methods

### 2.1. High-Performance Liquid Chromatography (HPLC) Analysis of MHT

HPLC analysis of the seven characteristic constituents in MHT was performed using a Shimadzu Prominence LC-20A series HPLC system (Kyoto, Japan) coupled with a photodiode array detector and LabSolutions software (Version 5.54 SP3, Shimadzu, Kyoto, Japan). A Waters Sun Fire C_18_ analytical column (250 × 4.6 mm, 5 *μ*m; Milford, MA, USA) as the stationary phase, maintained at 40°C, was used for the separation of the main components. The mobile phases consisted of 0.1% (v/v) trifluoroacetic acid in distilled water (A) and acetonitrile (B). The elution gradient used for chromatographic separation was as follows: 10%–60% B for 30 min, 60%–100% B for 30–40 min, 100% B for 40–45 min, 100%–10% B for 45–50 min, and 10% B for 50–60 min. The flow rate was set to 1.0 mL/min, and the injection volume was 10 *μ*L.

### 2.2. L-NAME Induction of Hypertension in Rats

Male Sprague-Dawley rats weighing 180–200 g were acclimatized for 7 days under a 12 h light-dark cycle. After which, the rats were randomly divided into five experimental groups: (1) control (*n* = 8); (2) L-NAME (40 mg/kg/day, *n* = 8); (3) L-NAME + Olmetec^®^ (OMT, angiotensin-II receptor type II blocker, 10 mg/kg/day, per os (p.o.)); (4) L-NAME + MHT (50 mg/kg/day, p.o.); and (5) L-NAME + MHT (100 mg/kg/day, p.o.). All L-NAME groups were administered L-NAME in drinking water for 5 weeks. Each group composed of 8 rats and all L-NAME-induced hypertensive groups were administered L-NAME in the drinking water for 5 weeks. After 3 weeks treated with L-NAME, MHT or OMT was injected by oral administration daily to L-NAME-induced hypertensive rats for 2 weeks.

### 2.3. Measurement of Blood Pressure

Systolic blood pressure (SBP) and body weight were assessed weekly by tail-cuff plethysmography (MK2000; Muromachi Kikai, Tokyo, Japan) in a quiet and warm room. At least seven determinations were carried out for every cycle of measurement and an average of five cycles of measurement was used for final comparisons. In this study, rats with SBP > 160 mmHg were used.

### 2.4. Preparation of Aortic Rings

Animal procedures were performed in strict accordance with the National Institute of Health Guidelines for the Care and Use of Laboratory Animals. Animal procedures were approved by the Institutional Animal Care and Utilization Committee of Wonkwang University. Male Sprague Dawley rats (250–300 g) were purchased from Korean Experimental Animals Co. (Wanju, Korea). The thoracic aorta was separated rapidly from these rats, and the fat was removed from aortic tissue in ice-cold Krebs' solution (pH 7.4) containing 118.0 mM NaCl, 1.1 MgSO_4_, 4.7 KCl, 1.2 KH_2_PO_4_, 25.0 NaHCO_3_, 10.0 glucose, and 1.5 CaCl_2_. The thoracic aorta was cut into rings, approximately 3 mm in width, and the endothelial layer in some aortic rings was removed by rubbing. The relaxation response in the aortic rings with and without the endothelium, precontracted with phenylephrine (PE, 1 *μ*M), upon acetylcholine (Ach, 1 *μ*M) was measured.

### 2.5. Measurement of cGMP Levels in Rat Aorta

Rat aortic rings were incubated for 60 min in flasks containing 5 mL of Krebs solution placed in a shaking water bath at constant temperature (37°C), 95% O_2_, and 5% CO_2_. This was followed by incubation with 3-isobutyl-1-methylxanthine (IBMX, 1 *μ*M) and PE (1 *μ*M) for 5 mins. After the aortic rings were incubated with MHT in the presence and absence of modulators for 4 min, the reactions were stopped by freezing the tissues in liquefied N_2_. The tissues were homogenized with 0.1 M HCl and centrifuged at 13000 rpm for 15 min. The protein concentration was determined by Bradford assay using bovine serum albumin (BSA) as the standard. The cGMP level was measured by an equilibrated radioimmunoassay, as described previously [11], and results have been expressed as nanomoles of cGMP per milligram of protein (nmol/ml/mg protein).

### 2.6. Hematoxylin-Eosin (H&E) Staining of Aorta

The thoracic aorta tissues were fixed in 10% (v/v) formalin. Fixed tissue samples were dehydrated and embedded in paraffin and then placed on a poly-L-lysine-coated slide (Fisher Scientific, Pittsburgh, PA, USA). Thin sections (4 *μ*M) of the aortic tissue were stained with H&E stain for histopathological analysis. The length of intima-to-media was determined using AxioVision 4 imaging/archiving software (AxioVision 4, Carl Zeiss, Germany).

### 2.7. Immunohistochemical (IHC) Analysis of the Aorta

Paraffin-embedded sections were immunostained using Invitrogen's Histostain^®^ Plus Broad Spectrum kit (Novex^®^, CA, USA). The slides were immersed in 3% hydrogen peroxide for 10 min at room temperature and incubated with primary antibodies against eNOS (1 : 200, Santa Cruz, CA, USA) and ET-1 (1 : 200, Santa Cruz, CA, USA) in humidified chambers overnight at 4°C. All slides were then incubated with a biotinylated secondary antibody for 2 h at room temperature. Peroxidase activity was visualized using 3,3′-diaminobenzidine (Novex^®^, CA) substrate-chromogen system and counterstained with hematoxylin (Zymed, CA, USA). ImageJ (NIH, Bethesda, MD, USA) was used to quantify protein expression.

### 2.8. Measurement of Plasma Biomarker

Blood samples were collected in test tubes containing ethylenediaminetetraacetic acid and centrifuged at 990 × g for 20 min at 4°C. Plasma samples were frozen at −70°C until further use. Plasma levels of albumin, BUN, and creatinine were measured using a commercial kit (Arkray Inc., Kyoto, Japan).

### 2.9. Measurement of Osmolality and Urinary Albumin

Rats from each group were maintained in separate metabolic cages during the experimental period, allowing for the quantitation of urine volume and water intake. Urine samples were collected on a day in the last week of the experimental period to determine osmolality and urine albumin levels. Osmolality was measured using an advanced cryomatic osmometer (Advanced Instruments, Norwood, MS).

### 2.10. Western Blot Analysis

Thoracic tissue homogenates (40 *μ*g of protein) were separated by 10% sodium dodecyl-polyacrylamide gel electrophoresis and transferred to a nitrocellulose membrane paper. Blots were then blocked with 5% BSA in Tris-buffered saline for 1 h and incubated with the appropriate primary antibody at dilutions recommended by the supplier. The membrane was then washed and incubated with the appropriate horseradish peroxidase-conjugated secondary antibody for 1 h. The bands were visualized using enhanced chemiluminescence (Amersham, Buckinghamshire, UK). Protein expression levels were determined by analyzing the signal intensity using a ChemiDoc image analyzer (Bio-Rad, Hercules, CA, USA).

### 2.11. Statistical Analysis

The multiple experimental groups were compared using repeated-measures analysis of variance followed by Bonferroni's multiple-comparison test. Two-group comparisons were performed using Student's unpaired *t*-test. Statistical significance was defined as *p* < 0.05. The results are presented as mean ± standard error of mean.

## 3. Results

### 3.1. HPLC Analysis of MHT

**Figure 1:**
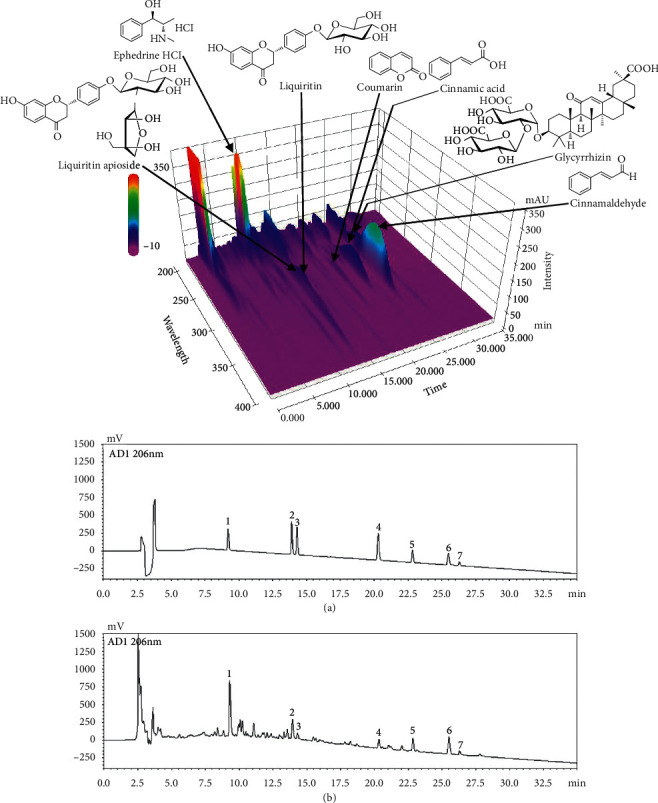
Representative HPLC chromatograms of standard solution (a) and Ma Hwang Tang decoction (b) measured at UV wavelength 206 nm. Ephedrine HCl (1), liquiritin apioside (2), liquiritin (3), coumarin (4), cinnamic acid (5), cinnamaldehyde (6), and glycyrrhizin (7).

HPLC was used to simultaneously detect the seven characteristic components of MHT. Each compound in the MHT was identified based on the retention time and UV spectra of the reference standard. Seven components, namely, ephedrine HCl, liquiritin apioside, liquiritin, coumarin, cinnamic acid, cinnamaldehyde, and glycyrrhizin, were eluted at 9.24, 13.94, 14.20, 20.30, 22.82, 25.50, and 26.31 min, respectively, and their concentrations in MHT measured using an optimized analytical assay were 2.68, 1.08, 0.74, 0.12, 0.09, 0.21, 0.36, and 1.60 mg/freeze-dried g, respectively (Figure 1).

### 3.2. MHT-Induced Vasodilation and cGMP Production

**Figure 2:**
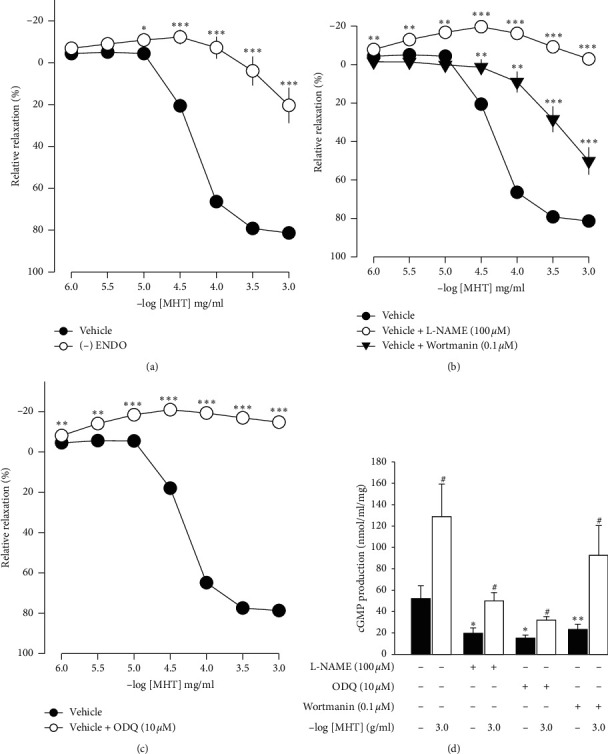
Effect of MHT on vasodilation and cGMP production. Presented vascular relaxation response to MHT in aorta with endothelium or without (a) Presence of L-NAME (100 *μ*M) or wortmannin (0.1 *μ*M) (b). Presence of ODQ (10 *μ*M) (c). The cGMP production response to MHT with L-NAME (100 *μ*M), ODQ (10 *μ*M), or wortmannin (0.1 *μ*M). MHT, Ma Huang Tang; cGMP: guanosine 3′,5′-cyclic monophosphate; L-NAME: N^G^-nitro-L-arginine methylester; and ODQ: 1H-[1,2,4]-oxadiazolo-[4,3-*α*]-quinoxalin-1-one. Values are expressed as mean ± S.E. (*n* = 5 per group). ^*∗*^*p* < 0.05, ^*∗∗*^*p* < 0.01, and ^*∗∗∗*^*p* < 0.001 versus vehicle: #*p* < 0.05 versus L-NAME, ODQ, or wortmannin.

In endothelium-intact thoracic aorta rings, MHT induced vascular relaxation in a dose-dependent manner. However, MHT-induced vasodilation was blocked in the endothelium-removed thoracic aorta (Figure 2(a)). MHT-induced vasodilation was inhibited in vascular tissue treated with wortmannin, which is a nonselective PI3K inhibitor, compared to untreated vascular tissue. MHT-induced vasodilation was completely abolished by pretreatment with 100 *μ*M L-NAME (Figure 2(b)). We used ODQ, a sGC inhibitor, to understand the mechanism of MHT-induced vasodilation. Pretreatment of vascular tissues with 10 *μ*M ODQ completely abolished MHT-induced vascular relaxant response of the thoracic aorta (Figure 2(c)). We found that MHT treatment resulted in an increase in cGMP production compared to that of the control. However, pretreatment with L-NAME, ODQ, or wortmannin in vascular tissues completely blocked the MHT-induced increases in cGMP production. When MHT was administered to aortic tissues pretreated with L-NAME, ODQ, or wortmannin, there was a significant increase in cGMP production (Figure 2(d)).

### 3.3. MHT Decreased SBP in L-NAME-Induced Hypertensive Rats

**Figure 3:**
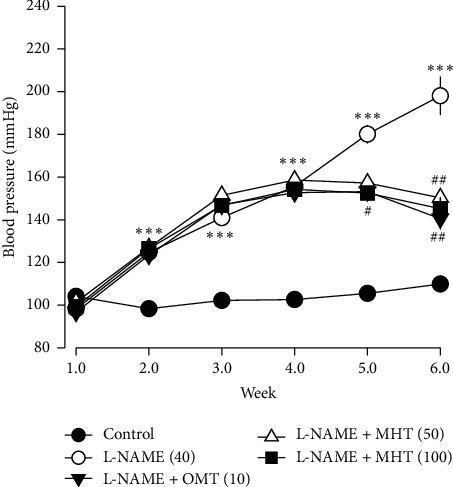
Effect of MHT on blood pressure. Presented systolic blood pressure measured by tail-cuff method (mmHg). MHT: Ma Huang Tang; OMT: Olmetec. Values are expressed as mean ± S.E. (*n* = 8 per group). ^*∗∗∗*^*p* < 0.01 versus control: ^#^*p* < 0.05, ^##^*p* < 0.01 versus L-NAME.

The mean SBP of L-NAME treated rats was 198 ± 8.9 mmHg. Hypertensive rats in the MHT treatment group exhibited significantly lower SBP than those that were treated with L-NAME alone (Figure 3).

### 3.4. MHT Restored Vascular Function in L-NAME-Induced Hypertensive Rats

**Figure 4:**
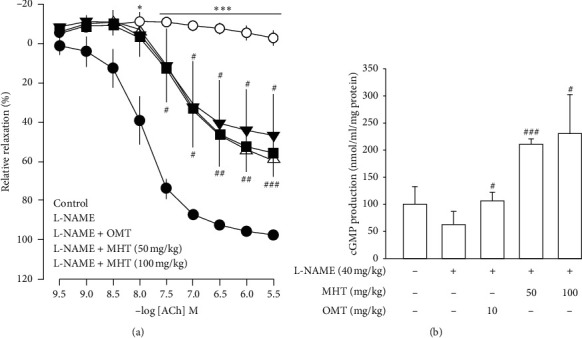
Effect of MHT on vascular relaxation and cGMP level in L-NAME model. (a) Vascular relaxation response to acetylcholine (Ach) in thoracic aorta of L-NAME model. (b) The cGMP production level in the thoracic aorta of control and L-NAME groups. MHT: Ma Huang Tang; cGMP: guanosine 3′,5′-cyclic monophosphate; OMT: Olmetec. Values are expressed as mean ± S.E. (*n* = 3 per group). ^*∗*^*p* < 0.05, ^*∗∗∗*^*p* < 0.001 vs. control: ^#^*p* < 0.05, ^##^*p* < 0.01, ^###^*p* < 0.001 vs. L-NAME.

Vasodilatory responses to ACh were significantly impaired in the L-NAME-induced hypertensive rats compared to that in the control group. However, the responses evoked by Ach were rescued by MHT treatment in a dose-dependent manner compared with that in the L-NAME hypertensive rats (Figure 4(a)). The cGMP level in the aorta of L-NAME rats was lower than that in control rats, but there was no significant. cGMP production in the MHT and OMT groups treated in L-NAME hypertensive rats was increased compared with that in the L-NAME group and control group (Figure 4(b)).

### 3.5. MHT Regulated Expression of Vascular Factors in L-NAME Hypertensive Rats

**Figure 5:**
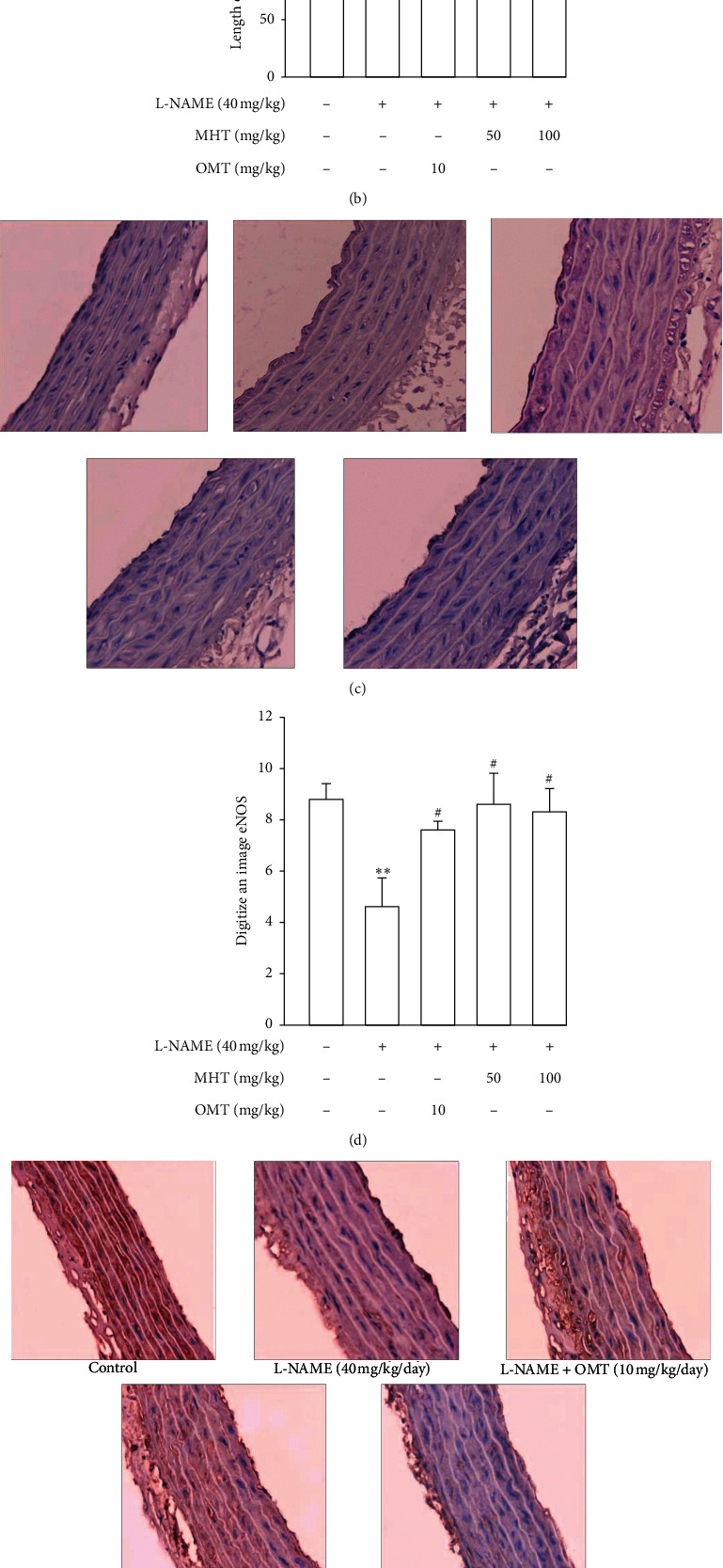
Effect of MHT on aorta morphology, eNOS, and ET-1 immunoreactivity in aortic tissues. (a) Representative microscopic photographs in aorta of control and L-NAME groups were stained with hematoxylin and eosin. (b) Numerical value of the length of tunica intima-media in aorta of L-NAME model. (c) eNOS immunoreactivity in thoracic aorta of L-NAME-induced hypertensive rats. (d) Digitization of eNOS expression. (e) ET-1 immunoreactivity in thoracic aorta of L-NAME-induced hypertensive rats. (f) Digitization of ET-1 expression. MHT: Ma Huang Tang; eNOS: endothelial nitric oxide synthase; ET-1: endothelin-1; OMT: Olmetec. Values are expressed as mean ± S.E. (*n* = 3 per group). ^*∗∗*^*p* < 0.01, ^*∗∗∗*^*p* < 0.001 versus control: ^#^*p* < 0.05, ^##^*p* < 0.01, and ^###^*p* < 0.001 versus L-NAME.

**Figure 6:**
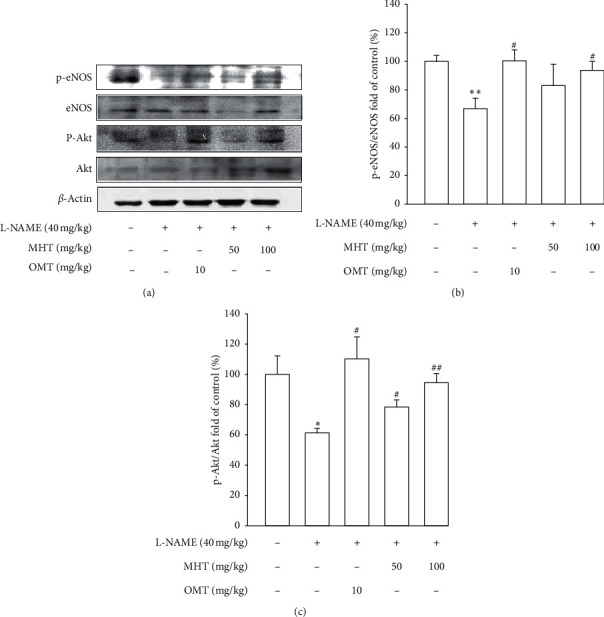
Effect of MHT on the expression of eNOS and Akt phosphorylation by western blot analysis. MHT: Ma Huang Tang; eNOS: endothelial nitric oxide synthase; and OMT: Olmetec. Values are expressed as mean ± S.E. (*n* = 3 per group). ^*∗*^*p* < 0.05, ^*∗∗*^*p* < 0.01 versus control: ^#^*p* < 0.05, ^##^*p* < 0.01 versus L-NAME.

Morphological staining showed that the L-NAME group had an increased thickness of intimal endothelial and medial layers compared to that of the control group. However, treatment with MHT in L-NAME hypertensive rats resulted in markedly decreased thickness of intimal endothelial and medial layers (Figures 5(a) and 5(b)). As shown in Figure 5(c), eNOS expression was suppressed in the aortic tissue of the L-NAME group compared to that in the control group. However, treatment with MHT (50 or 100 mg/kg/day) restored eNOS expression in the L-NAME group (Figures 5(c) and 5(d)). In contrast, aortic ET-1 expression was increased in the L-NAME hypertensive group and MHT treatment groups in L-NAME hypertensive rats lowered this compared to that in the L-NAME group (Figures 5(e) and 5(f)). To demonstrate the effect of MHT on blood vessels, thoracic tissues derived from the experimental rats were subjected to western blotting. In the L-NAME group, phospho-Akt1/2/3 and phospho-eNOS expression decreased compared with that of the control, and treatment with MHT rescued this alteration in the expression of phosphorylated Akt/1/2/3 and eNOS (Figure 6).

### 3.6. MHT Ameliorated Renal Dysfunction in L-NAME-Induced Hypertensive Rats

The plasma levels of BUN and creatinine were remarkably upregulated in the L-NAME group compared with those in the control group. However, the levels of BUN and creatinine in serum were downregulated and albumin was upregulated in the MHT and OMT treatment groups compared to those in the only L-NAME treatment group (Table 1). There was no significant difference in water intake and urine volume between the L-NAME and control groups. MHT treated in L-NAME-induced hypertensive rats also had no effect on water intake and urine volume compared to that in the L-NAME group. The level of osmolality was lower in the L-NAME-induced hypertension group than that in the control group. However, the MHT-treated group in L-NAME-induced hypertensive rats showed a significantly increased osmolality compared to that in the L-NAME group (Table 2). Urinary albumin levels were higher in the L-NAME group than those in the control group. MHT treatment significantly lowered the urinary albumin level in L-NAME-treated rats compared to that in rats treated with L-NAME alone (Table 2).

## 4. Discussion

MHT has long been used in Korean traditional medicine for the treatment of various diseases and has been previously reported to exhibit protective effects against cough and asthma [8, 9]. However, little is known about the mechanisms that underlie the pharmacological activity of MHT in ethnomedicine. Ephedrine is the most abundant alkaloid found in MHT and has a chemical structure comparable to that of amphetamines, ephedrine. It works directly or indirectly by stimulating the alpha adrenergic and beta adrenergic receptors and trace amine-associated receptors [12, 13]. This receptor activation by ephedrine has been reported extensively and is probably involved in the severe cardiovascular side effects of the Ma Huang decoction (MHD). Interestingly, the contractile response triggered by MHT is much higher than that of a combination of MHT and other components in the MHD. This confirms that the interplay of the various components in the MHD may mitigate the side effects of the MHD [10]. Thus, this study attempted to determine whether MHT, not MHD, would have a beneficial effect on blood pressure-associated hypertension.

Vascular endothelium is known to produce NO, which is an important regulator of vascular tone. NO diffuses out of the endothelium and a fraction of it enters the underlying vascular smooth muscle cells, where it binds to and activates sGC in vascular smooth muscle cells and catalyzes the generation of cGMP, a second messenger, leading to vasorelaxation [14]. We found that MHT did not induce vascular relaxation in endothelium-removed rat thoracic aortic rings, and L-NAME and ODQ significantly inhibited MHT-induced vascular relaxation in endothelium-intact aortic rings. Furthermore, MHT increased cGMP production in aortic smooth muscle, but pretreatment with ODQ and L-NAME blocked MHT-induced increase in cGMP production. These results suggest that MHT induces endothelium-dependent activation of the NO-cGMP pathway and vascular relaxation.

High SBP is a major risk factor for heart disease, stroke, coronary heart disease, and kidney disease [15]. Pharmacologic treatment for hypertension is effective; however, long-term pharmacological therapy can have adverse effects [16]. On the other hand, herbal medicines are known to have few side effects, but further studies are needed to demonstrate the effect of oriental medicine on hypertension. Therefore, in this study, we investigated the effect of MHT on ameliorating symptoms of hypertension in rats treated with L-NAME. Recently, many investigators have reported that intravenous administration of L-NAME has been associated with dose-dependent increases in SBP [17, 18].

The present study showed that treatment with 50 or 100 mg/kg/day MHT lowered blood pressure in L-NAME-induced hypertensive rats. Endothelial function is an important regulator of vascular relaxation. Hypertension induces endothelial dysfunction, which is key to the pathogenesis of coronary heart disease and also increases the risk of developing it. [19]. ACh induced a concentration-dependent dilation of the aorta in control rats. ACh binds to specific endothelial receptors and causes the activation of endothelial NO synthesis, resulting in NO release [20]. Vasodilation in response to ACh stimulation was significantly inhibited in L-NAME hypertensive rats compared to that in normotensive MHT-treated L-NAME hypertensive rats. Treatment with OMT, an ACE inhibitor, also significantly improved vasodilation responses in L-NAME-induced hypertensive rats. NO produced by endothelial cells promotes vascular relaxation by activating guanylate cyclase and increasing cGMP production in vascular smooth muscle [21]. The aortic levels of cGMP were decreased in the L-NAME hypertensive group compared with that in the control group; however, cGMP production in the thoracic aorta was restored after treatment with MHT. Thus, our results demonstrate that MHT-induced vascular dilation is related to decreased blood pressure in L-NAME-induced hypertensive rats.

**Figure 7:**
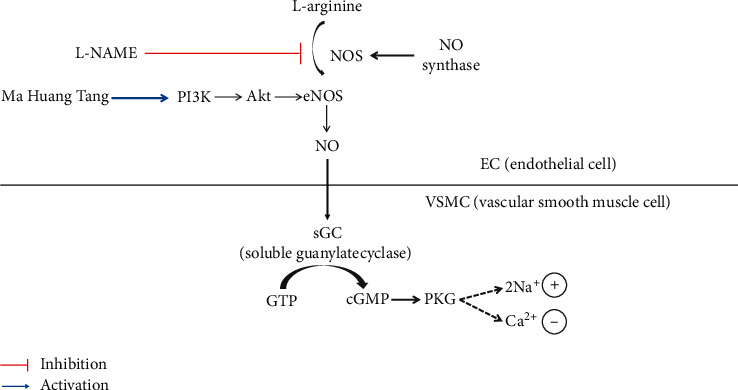
Summary of MHT action in L-NAME-induced hypertensive rats. L-NAME inhibited NO production; therefore, Akt, eNOS, and cGMP activation was decreased. Meanwhile, MHT activated PI3K/Akt/eNOS signaling; as a result, cGMP production was increased. MHT: Ma Huang Tang; eNOS: endothelial nitric oxide synthase; cGMP: guanosine 3′,5′-cyclic monophosphate; and OMT: Olmetec.

Several studies have shown that lowering blood pressure and endothelial function are associated with an increase in eNOS reactivity, which in turn increases NO production and vasodilation [22, 23]. Immunohistochemical staining analysis showed that eNOS was weakly expressed in the thoracic aorta of the L-NAME hypertensive group, but this expression was markedly increased in the MHT group. On the other hand, the expression of ET-1 was lower in the MHT group than that in the L-NAME hypertensive group. ET-1 is a potent endothelium-derived vasoconstrictor that causes a prolonged increase in vascular tone [24]. It has also been identified as an important vasoactive substance associated with pathophysiological conditions such as hypertension, ischemic heart disease, and congestive heart failure [25, 26]. These results suggest that MHT ameliorates ACh-induced vascular relaxation by improving the eNOS/cGMP pathway in the L-NAME hypertensive animal model (Figure 7).

There have been many reports suggesting that BUN and creatinine clearance levels are generally considered as prognostic markers of renal function. Moreover, systemic development of hypertension and renal vasoconstriction has been observed upon chronic blockade of endogenous NO synthesis; that is, chronically NO-blocked rats developed proteinuria and glomerular sclerotic injury [27, 28]. Hence, urinary albumin, BUN, and creatinine clearance levels were determined to evaluate the effect of MHT. In this study, BUN and creatinine clearance increased significantly in L-NAME hypertensive rats, whereas MHT suppressed the upregulation of BUN and creatinine clearance. Moreover, osmolality is upregulated by MHT compared to that in L-NAME hypertensive rats. These results suggest that MHT ameliorates renal dysfunction in L-NAME-induced hypertension rat model. Additional experiments including preclinical and clinical study are required to demonstrate the safety and LD50 value of MHT in hypertension.

## 5. Conclusions

This study provides evidence for the therapeutic role of MHT in suppressing blood pressure and renal dysfunction in rats with L-NAME-induced hypertension, via the activation of the vascular NO/cGMP system. Furthermore, our results indicate that MHT may play an important role in the regulation of blood pressure and renal function in experimental hypertensive model.

## Figures and Tables

**Table 1:** Effect of MHT on the BUN, albumin, and creatinine in the serum of L-NAME-induced hypertensive rats.

	Cont.	L-NAME	OMT 10 (mg/kg/day)	MHT 50 (mg/kg/day)	MHT 100 (mg/kg/day)
Albumin (g/dl)	3.4 ± 0.05	3.02 ± 0.12^*∗∗*^	3.78 ± 0.1^###^	3.4 ± 0.1^##^	3.55 ± 0.1^##^
BUN (mg/dl)	14.7 ± 0.9	51 ± 13.0^*∗∗*^	22.9 ± 0.42^##^	21 ± 1.4^###^	20.3 ± 1.6^##^
Creatinine (mg/dl)	0.7 ± 0.05	1.21 ± 0.2^*∗∗∗*^	1.15 ± 0.14	0.8 ± 0.1^###^	0.77 ± 0.1^###^

Serum albumin, BUN, and creatinine were measured by using kits as described in the methods. Cont.: control; MHT: Ma Huang Tang; and OMT: Olmetec. The data of values shows mean ± S.E. (*n* = 8 per group). ^*∗∗*^*p* < 0.01, ^*∗∗∗*^*p* < 0.001 versus control; ^##^*p* < 0.01, ^###^*p* < 0.001 versus L-NAME.

**Table 2:** Effect of MHT on the body weight, water intake, urine volume, and osmolality in urine of L-NAME-induced hypertension rats.

	Cont.	L-NAME	OMT 10 (mg/kg/day)	MHT 50 (mg/kg/day)	MHT 100 (mg/kg/day)
Water intake (ml/day)	22.4 ± 1.3	20.9 ± 4.4	25.3 ± 3.9	18.06 ± 9.4	23.7 ± 5.0
Urine volume (g/23 h)	32.9 ± 1.0	42.4 ± 5.9	32.9 ± 2.5	32.2 ± 0.4	35.7 ± 2.2
Osmolality (mOsm/kg H_2_O)	2262.7 ± 110	795 ± 79.9^*∗∗∗*^	1932.6 ± 82.3^###^	1510.5 ± 19.5^##^	1720.8 ± 94.6^###^
Albumin (g/dl)	0.473 ± 0.01	0.60 ± 0.05^*∗*^	0.56 ± 0.06	0.433 ± 0.07^#^	0.269 ± 0.02^###^

Water intake and urine samples were acquired for 1 day at last week of experiment. Cont.: control; MHT: Ma Huang Tang; and OMT: Olmetec. The data of values shows mean ±  S.E.(*n* = 4 per group). ^*∗*^*p* < 0.05; ^*∗∗*^*p* < 0.001 versus control; ^#^*p* < 0.05, ^##^*p* < 0.01, and ^###^*p* < 0.001 versus L-NAME.

## Data Availability

The data used to support the findings of this study are included within the article.
